# Association between light rare earth elements in maternal plasma and the risk of spontaneous preterm birth: a nested case-control study from the Beijing birth cohort study

**DOI:** 10.1186/s12940-023-01027-1

**Published:** 2023-10-23

**Authors:** Junxi Chen, Aili Wang, Hang An, Weiling Han, Junhua Huang, Wei Zheng, Lailai Yan, Zhiwen Li, Guanghui Li

**Affiliations:** 1 Institute of Reproductive and Child Health, National Health Commission Key Laboratory of Reproductive Health, Peking University, Beijing, 100191 PR China; 2 Department of Epidemiology and Biostatistics, School of Public Health, Peking University, Beijing, 100191 PR China; 3 Division of Endocrinology and Metabolism, Department of Obstetrics, Beijing Obstetrics and Gynecology Hospital, Beijing Maternal and Child Health Care Hospital, Capital Medical University, Beijing, 100026 PR China; 4 Beijing Luhe Hospital, Capital Medical University, Beijing, 101100 PR China; 5 Department of Laboratorial Science and Technology, School of Public Health, Peking University, Beijing, 100191 PR China

**Keywords:** Rare earth elements, Spontaneous preterm birth, Nested case-control study, Preterm premature rupture of membranes, Spontaneous preterm labor

## Abstract

**Background:**

Parental exposure to rare earth elements (REEs) could increase the risk of premature rupture of membranes, a major cause of spontaneous preterm birth (SPB). In addition, different subtypes of SPB, such as spontaneous preterm labor (SPL) and preterm premature rupture of membranes (PPROM), may have different susceptibility to environmental exposure. Therefore, we investigated the potential associations between REE exposure in different trimesters and SPB and its subtypes.

**Methods:**

A nested case-control study was performed. We included 244 women with SPB as cases and 244 women with full-term delivery as controls. The plasma concentrations of light REEs were measured in the first and third trimesters. Logistic regression was used to analyze the associations between single REE levels and SPB, and Bayesian kernel machine regression (BKMR) was used to analyze the mixed-exposure effect.

**Results:**

Exposure to light REEs was associated with SPB and its subtypes only in the third trimester. Specifically, the intermediate- and highest-tertile concentration groups of La and the highest-tertile concentration group of Sm were associated with an increased risk of SPL, with adjusted odds ratios (AORs) of 2.00 (95% CIs: 1.07–3.75), 1.87 (95% CIs: 1.01–3.44), and 1.82 (95% CIs: 1.00–3.30), respectively. The highest-tertile concentration group of Pr was associated with an increased risk of PPROM, with an AOR of 1.69 (95% CIs: 1.00–2.85). Similar results were also found in BKMR models.

**Conclusions:**

La and Sm levels in plasma may be associated with the risk of SPL, and Pr levels in plasma may be associated with the risk of PPROM.

**Supplementary Information:**

The online version contains supplementary material available at 10.1186/s12940-023-01027-1.

## Introduction

Preterm birth, which is widely defined as all births at < 37 gestational weeks, is the leading cause of death in children aged under 5 years worldwide and may increase the risk of long-term morbidities [1, 2]. Approximately 5–7% of neonates, or one million neonates per year, are affected by preterm birth in China [1–3]. Preterm birth can be classified as spontaneous preterm labor (SPL), preterm premature rupture of membranes (PPROM), and provider-initiated preterm birth (induced labor or elective cesarean delivery for maternal and fetal indications) [4, 5]. SPL and PPROM are both designated spontaneous preterm birth (SPB) [4]. The causes of SPB are unclear, although some risk factors have been reported, including infection, poor nutritional status, and maternal age [4]. Some studies have found that exposure to certain trace elements, such as copper, manganese, mercury, and lead, may be related to SPB [6–9]. Liu et al. found that parental exposure to rare earth elements (REEs) could increase the risk of premature rupture of membranes, a major cause of SPB [10]. However, to the best of our knowledge, there is no evidence of associations between REE exposure and SPB.

REEs include scandium, yttrium, and 15 lanthanides: lanthanum (La), cerium (Ce), praseodymium (Pr), neodymium (Nd), promethium (Pm), samarium (Sm), europium (Eu), gadolinium, terbium, dysprosium, holmium, erbium, thulium, ytterbium and lutecium [11]. They are generally classified as light REEs(La, Ce, Pr, Nd, Pm, Sm, and Eu) and heavy REEs(other elements except for scandium) [11, 12]. REEs are widely used in electronics, manufacturing, medicine, and agriculture [13]. People can be exposed to them through water, food, the atmosphere, and through some medical tests [13, 14]. China has the largest reserves of REEs and contributes more than 90% of the global supply [12, 13]. Mining in China has resulted in higher REE concentrations in environmental components (i.e., soil, water, and atmosphere) than in other countries [15, 16]; this may be worsened by nearby coal burning and traffic emissions [16]. It has also been found that REE concentrations in food are higher in China than in other countries [17, 18]. Thus, the levels of environmental exposure to and the health risks of REEs in China are likely high.

Previous studies have found that environmental REE exposure can lead to excess health risks in pregnancy. With the exception of a premature rupture of membranes, Liu et al. also found that parental REE exposure may be associated with neonatal thyroid-stimulating hormone levels [10, 19]. Two case-control studies conducted in Shanxi Province, China, found that REE exposure increased the risk of birth defects, including orofacial clefts and neural tube defects [20, 21]. Li et al. found that high La exposure may increase the failure risk of in vitro fertilization-embryo transfer [22]. Previous in vivo and in vitro experiments have demonstrated that high REE exposure can cause increased oxidative stress levels, inflammatory cytokine expression, cytogenetic toxicity, neurotoxicity, and liver toxicity [23–25]. Oxidative stress and inflammation are usually thought to be detrimental factors in preterm birth pathology [26, 27].

We hypothesized that high REE exposure could increase the risk of SPB. In addition, previous studies have suggested that SPL and PPROM may be caused by different pathways and could have different susceptivity to environmental exposure [28, 29]. Hence, we further explored the associations between REE exposure and SPL and PPROM. Only light REEs were analyzed due to the low detection rate of heavy REEs.

## Methods

### Study design and participants

**Fig. 1:**
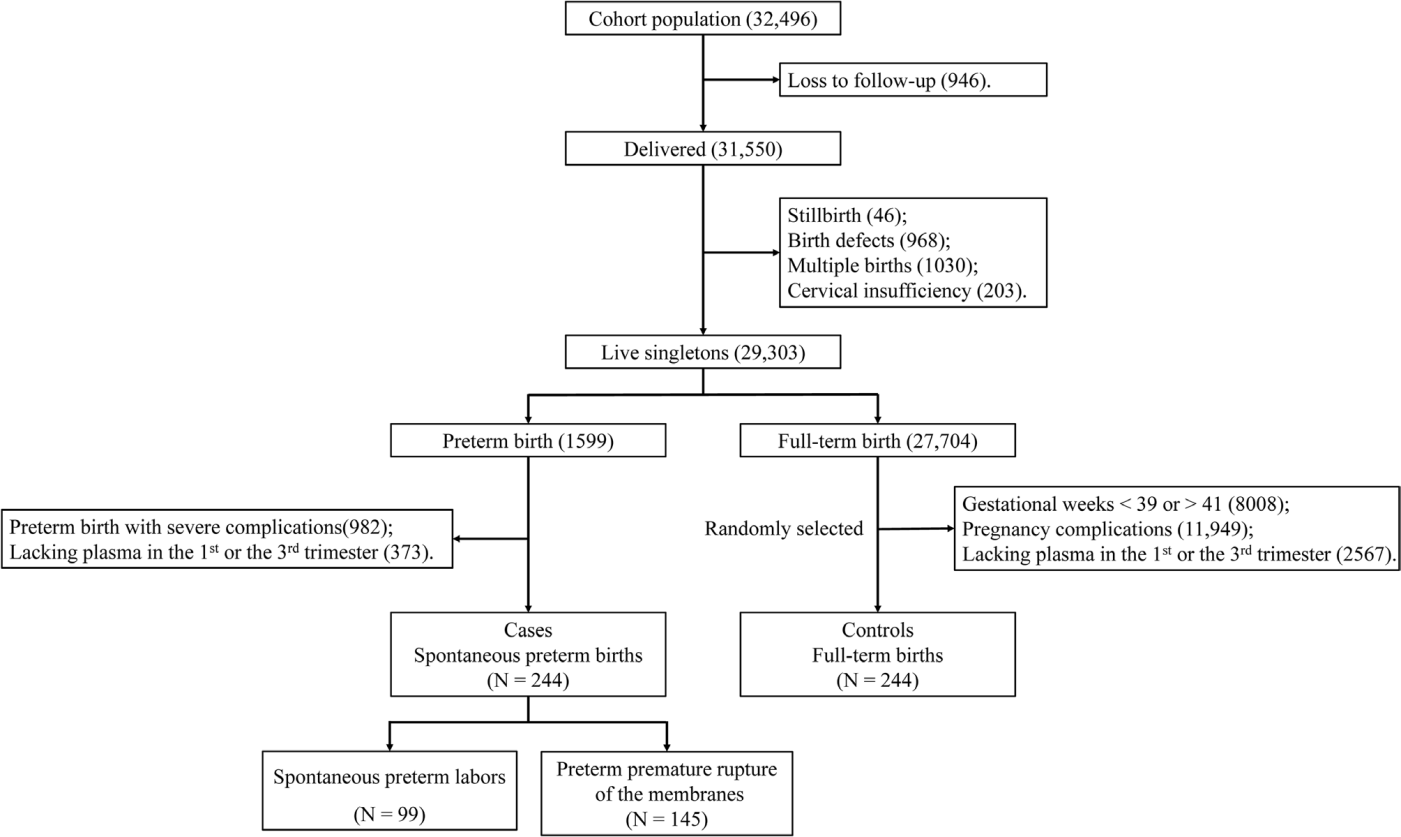
Selection of cases and controls from the cohort

This nested case-control study was based on the Beijing Birth Cohort Study (registered on the Chinese Clinical Trial Registry: ChiCTR2200058395) at Beijing Obstetrics and Gynecology Hospital. Pregnant women were recruited into the cohort if they consented, and the following inclusion criteria were met: 18–44 years old, planned to accept routine prenatal examinations and delivery at Beijing Obstetrics and Gynecology Hospital, first parental examination was at < 14 gestational weeks, and provided signed informed consent. From July 2018 to October 2020, 32,496 pregnant women were recruited. Maternal sociodemographic data were collected by questionnaires when recruiting. This study was approved by the Ethics Committee of the Beijing Obstetrics and Gynecology Hospital (approval number: 2018-KY-009-01), and all participants provided signed informed consent. Pregnant women were excluded if they were lost to follow-up (n = 946), had a stillbirth (n = 46), had a baby with birth defects (n = 968), had multiple births (n = 1030), or had cervical insufficiency (n = 203) (Fig. 1). Gestational weeks were estimated counting from the women’s last menstrual period and were verified by ultrasonography in the first trimester. A live birth at ≥ 28 and < 37 gestational weeks was diagnosed as preterm birth according to the guideline for preterm birth in China [30]. A total of 29,303 women remained, including 1599 preterm births and 27,704 full-term births.

Of all preterm births, 982 were induced labor or elective cesarean delivery for severe complications (i.e., severe preeclampsia, eclampsia, HELLP syndrome, cholestasis, fetal distress, placental abruption, placenta previa and acute appendicitis) and 373 women lacked plasma samples in the first or the third trimester; these two groups were further excluded. The subtypes of SPB were defined based on the characteristics of the initiation of labor [5, 31, 32]. SPL was initiated by spontaneous contractions and cervical dilation, without premature rupture of membranes. PPROM was initiated by premature rupture of membranes. Of all full-term births, 22,524 women were further excluded due to their gestational weeks being < 39 or > 41, have being diagnosed with pregnancy complications (gestational hypertension, gestational diabetes mellitus, fetal growth restriction, and others), or lacking plasma samples in the first or the third trimester. Then, 244 full-term births (controls) were randomly selected at a ratio of 1:1. Thus, 244 cases (i.e., SPB) and 244 controls were included in our study. Among these, 99 women had SPL and 145 had PPROM.

### Laboratory analyses

In the first and third trimester, maternal blood was collected by healthcare workers into vacuum blood collection tubes lined with ethylenediaminetetraacetic acid (EDTA). After collection, the samples were centrifuged at 1680 g for 10 min within 1 h, and plasma was extracted from the supernatant, stored at − 80 ℃, and thawed before analysis. The plasma concentrations of REEs were measured via inductively coupled plasma-mass spectrometry (ICP-MS, ELAN DRC II; PerkinElmer, USA). Pm values were not analyzed in the lab due to its radioactivity. Quantitative analyses were conducted at the Central Laboratory of Biological Elements in Peking University Health Science Center, and experiments were performed following the China Metrology Accreditation protocols. The specific methods used are described in previous studies, and some instrument conditions are shown in Text S1 [22, 33]. Briefly, 0.1 mL plasma was spiked with 0.1 mL internal standard (rhenium, Re) and 1.8 mL 1% nitric acid, and then the mixture was analyzed via ICP-MS. Standard materials (National Standard Material Center in China, standard materials of rare earth elements in human hair, GBW09101a) were used to assess accuracy, and the measured values fit the reference values (Table S1). The method detection limits (MDLs) and method quantification limits are shown in Table S1. The detection rates of most light REEs were greater than 85%, except for Eu (Table S2). In the third trimester, only 243 controls were included because one plasma sample was not adequate for laboratory analyses.

### Statistical analysis

The characteristics of pregnant women in different groups were compared using the t-test, ANOVA test, and Pearson chi-square test. The concentrations of REEs below MDLs were replaced by 1/√2 of MDLs for the statistical analysis, except for correlation analysis. Due to the nonnormal distribution of the data, the concentrations of REEs were log(e)-transformed. The median, the lower quartile, and the upper quartile were used to describe the distribution of REEs. The Mann-Whitney U test and the Kruskal-Wallis test were used to compare concentrations among different groups. Spearman’s correlation coefficient was determined to assess correlations between REEs.

Then, the concentrations of REEs were classified into high, intermediate, and low exposure groups based on their tertiles. Eu was classified into low (< MDL) and high (≥ MDL) groups because of its low detection rate. Binary logistic regression was used to obtain the odds ratio (OR) and the corresponding 95% confidence intervals (CIs) of SPB. Multinomial logistic regression was used to obtain the ORs and 95% CIs of different subtypes of SPB (i.e., SPL and PPROM). Ethnicity, education level, age, pre-pregnancy BMI, parity, and family income were considered potential confounders based on previous studies [4, 34]. These were treated as categorical variables (the specific classification is shown in Table 1) and adjusted in logistic models to obtain the adjusted OR (AOR). We combined two age groups (20–24 and 25–29) into one (20–29), and two BMI groups (24–28 and ≥ 28) into one (≥ 24), considering that only one woman in the case group was < 25 years old and only three women in the control group were ≥ 28 kg/m^2^. Each element was modeled separately in logistic regression models. Statistical significance was defined as a two-tailed *P* < 0.05. Due to the high correlations between REEs and mixed exposure in general observations, Bayesian kernel machine regression (BKMR) was applied to verify the association between REE exposure and the risk of SPB and its subtypes [35]. Posterior inclusion probability (PIP) was used to assess the relative importance of each REE in BKMR. Due to the low detection rate of Eu, we excluded it from the BKMR to test the robustness of the results. Sensitivity analysis was performed in pregnant women without group B Streptococcus (GBS) infection, considering that this was an important risk factor for preterm birth [36, 37]. All statistical analyses were performed using R software (version 4.1.2; R Foundation for Statistical Computing, Vienna, Austria).

## Result

The characteristics of the pregnant women who were included in the final analysis are presented in Table 1. The mean age of all included women was 31.5 ± 3.9 years old. The ranges of gestational weeks were 33–36 in the case group and 39–41 in the control group. No women were smokers. Ethnicity, education level, family income, and GBS infection status were comparable between the case and control groups. However, age and BMI were higher in the case group. Larger proportions of multiparous and/or overweight women were found in the case group. The sampling time of plasma was comparable between the groups both in the first and third trimester. Similar results were also found in the SPL, PPROM, and control groups (Table S3).

**Table 1:** The characteristics of pregnant women in cases and controls

Characteristics ^a^	Case (n = 244)	Control (n = 244)	*P* ^b^
Age (year)			
Mean ± SD	31.9 ± 4.0	31.1 ± 3.8	0.018
20–29	76 (31.1)	85 (34.8)	0.128
30–34	101 (41.4)	111 (45.5)	
≥ 35	67 (27.5)	48 (19.7)	
BMI (kg/m^2^)			
Mean (SD)	22.1 ± 3.2	21.2 ± 2.4	< 0.001
Slim (BMI < 18.5)	29 (11.9)	29 (11.9)	0.003
Normal (18.5 ≤ BMI < 24)	154 (63.1)	183 (75.0)	
Overweight (BMI ≥ 24)	61 (25.0)	32 (13.1)	
Ethnicity			
Han	223 (91.4)	226 (92.6)	0.738
Others	21 (8.6)	18 (7.4)	
Education level			
Junior college and below	66 (27.0)	49 (20.1)	0.164
Undergraduate	123 (50.4)	140 (57.4)	
Postgraduate and above	55 (22.5)	55 (22.5)	
Family income (yuan/month)			
< 10,000	61 (25.0)	50 (20.5)	0.336
10,000–19,999	96 (39.3)	93 (38.1)	
≥ 20,000	87 (35.7)	101 (41.4)	
Parity			
Primiparous (= 1)	157 (64.3)	186 (76.2)	0.006
Multiparous (≥ 2)	87 (35.7)	58 (23.8)	
GBS infection			
Yes	9 (3.7)	15 (6.1)	0.295
No	235 (96.3)	229 (93.9)	
Smoke			
No	244 (100)	244 (100)	/
Infant sex			
Male	136 (55.7)	125 (51.2)	0.364
Female	108 (44.3)	119 (48.8)	
Sampling times (gestational weeks)			
First trimester	8.3 ± 1.6	8.3 ± 1.7	0.892
Third trimester	33.8 ± 0.9	33.8 ± 1.1	0.824

^a^: Data are presented as “mean ± SD” or “number (percentage)”

^b^: Statistical test by Pearson chi-square test when variables were categorized and t-test when variables were continuous

**Fig. 2:**
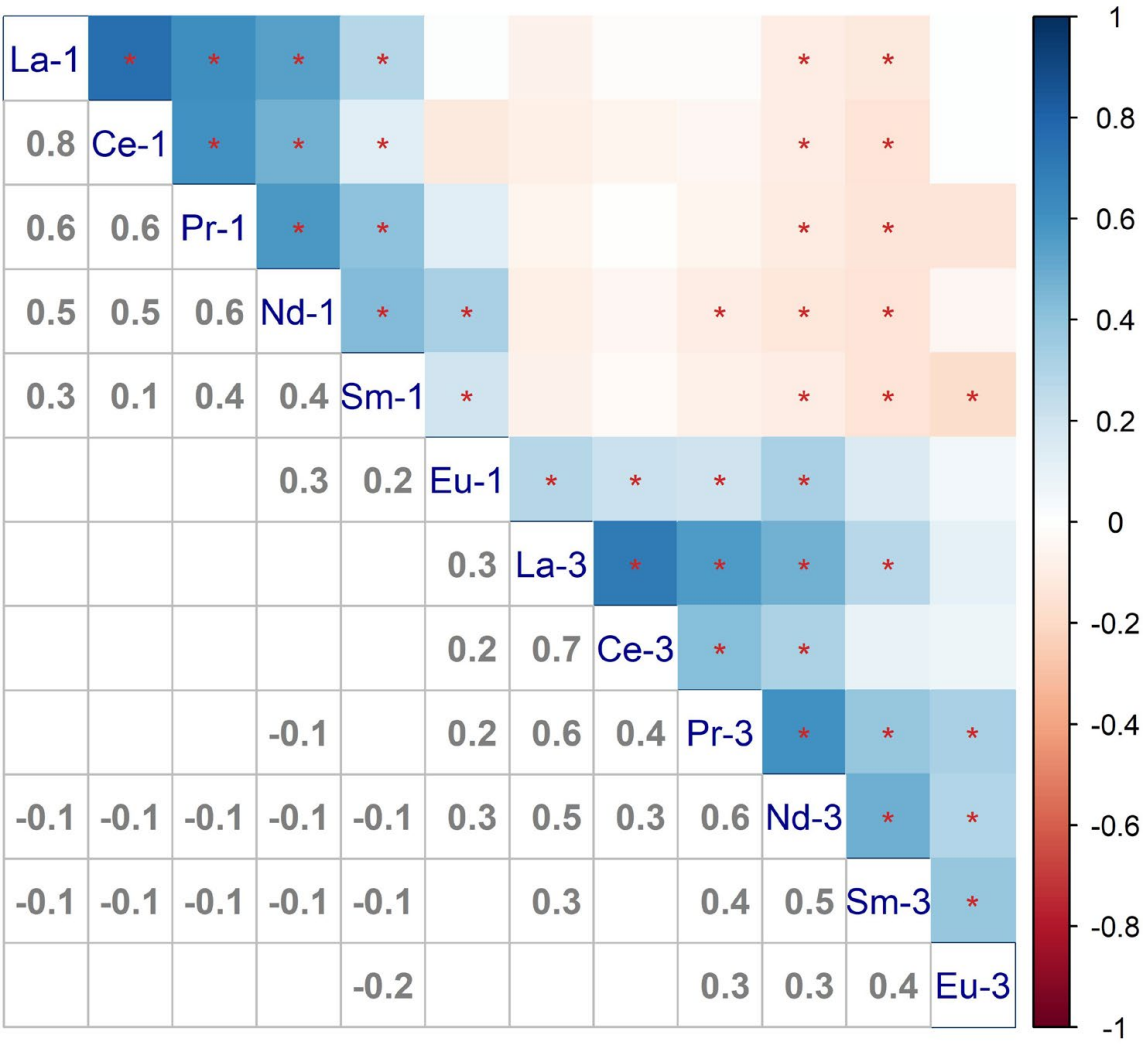
**Correlations among different elements during different trimesters.** -1: first trimester; -3: third trimester; *: *P* < 0.05; the lower-left part showed the correlation coefficients

La was the most common REE in plasma in our population. The median (quartiles) concentrations of La in our population were 0.071 (0.054–0.088) ng/mL in the first trimester and 0.073 (0.059–0.096) ng/mL in the third. Most REEs were positively related to each other in both the first and third trimesters, except for Eu (Fig. 2). La and Ce were most positively related, with correlation coefficients ranging from 0.7 to 0.8. In different trimesters, while negative correlations were found among REEs, their correlation coefficients were relatively small. The concentrations of REEs in different groups are shown in Table 2. Unlike what we anticipated, the differences in REE concentrations were not statistically significant between the case and control groups. Similar results were also found among the SPL, PPROM, and control groups (Table S4).

**Table 2:** Comparison of median concentrations of rare earth elements between cases and controls

Trimester	Elements	Case (n = 244)	Control (n = 244 ^a^)	*P* ^b^
First	La	0.073 (0.059, 0.093) ^c^	0.073 (0.058, 0.099)	0.708
	Ce	0.118 (0.085, 0.159)	0.126 (0.091, 0.165)	0.347
	Pr	0.031 (0.023, 0.040)	0.030 (0.024, 0.038)	0.674
	Nd	0.163 (0.113, 0.220)	0.161 (0.115, 0.231)	0.882
	Sm	0.047 (0.021, 0.080)	0.048 (0.024, 0.076)	0.718
	Eu	0.014 (0.014, 0.016)	0.014 (0.014, 0.014)	0.527
Third	La	0.072 (0.055, 0.090)	0.070 (0.052, 0.088)	0.217
	Ce	0.103 (0.073, 0.143)	0.102 (0.071, 0.134)	0.234
	Pr	0.031 (0.024, 0.040)	0.030 (0.021, 0.037)	0.111
	Nd	0.179 (0.115, 0.245)	0.173 (0.118, 0.234)	0.453
	Sm	0.055 (0.029, 0.096)	0.049 (0.022, 0.091)	0.355
	Eu	0.014 (0.014, 0.026)	0.014 (0.014, 0.023)	0.485

^a^: In the third trimester, only 243 controls were included because one plasma sample was not adequate for laboratory analyses

^b^: Statistical test by the Mann-Whitney U test

^c^: Units: ng/mL; Data are presented as “median (lower quartile, upper quartile)”

**Fig. 3:**
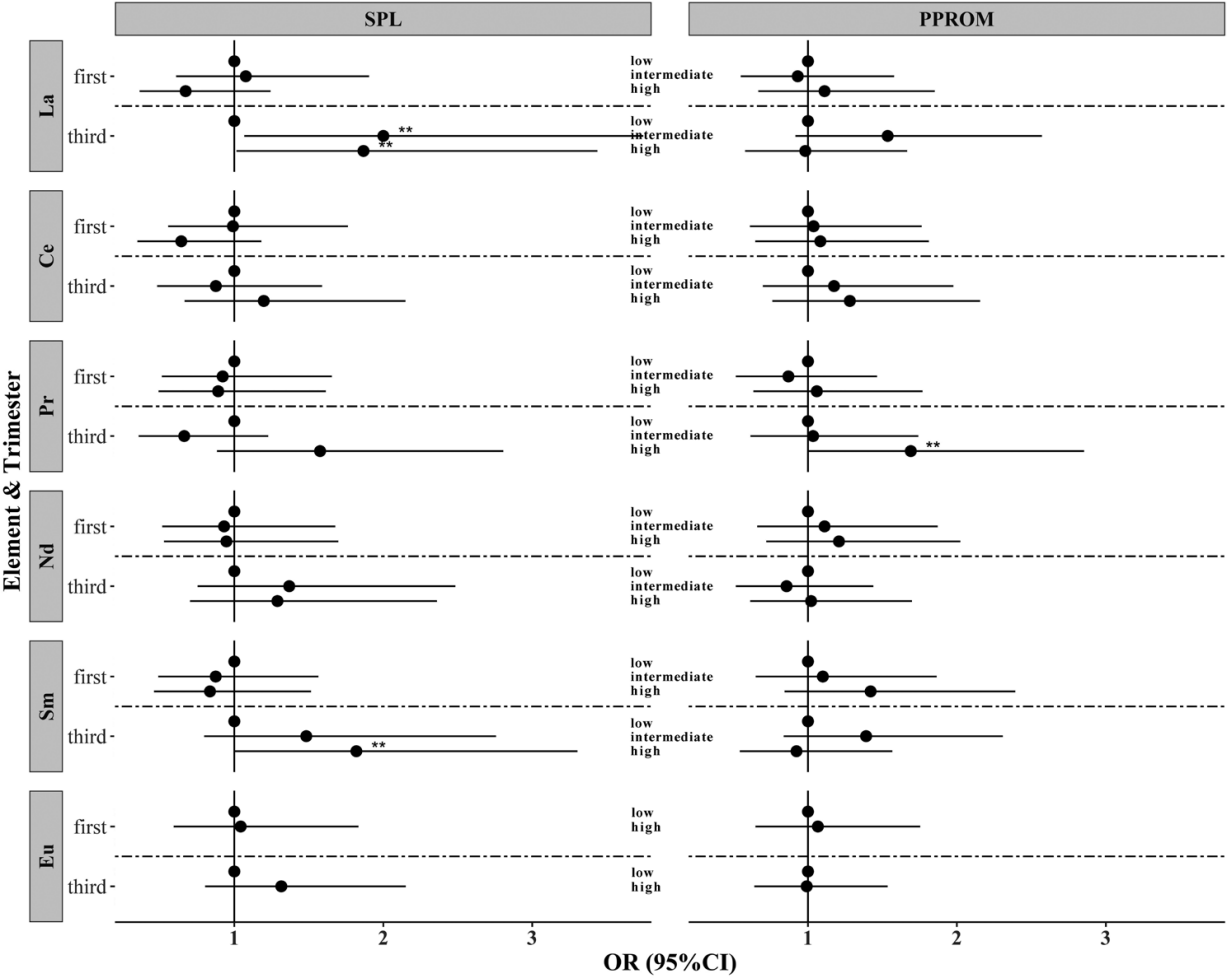
**Association between levels of rare earth elements in different trimesters and SPL and PPROM.** Adjusted for ethnicity, education level, age, BMI, parity, and family income; **: *P* < 0.05

Table 3 and S5 show the associations between REE exposure levels and the risk of SPB in the first and third trimesters. Overall, no significant association was found in the first trimester with or without adjustment for confounders (Table S5). For the third trimester, La and Pr were associated with SPB risk (Table 3). The intermediate exposure level for La significantly increased the risk of SPB, with an AOR of 1.70 (95% CIs: 1.07–2.68, *P* = 0.024). However, no significant association was found between the high La exposure level and the risk of SPB (*P* = 0.281). Compared to the low Pr exposure level, the high exposure level significantly increased the risk of SPB, with an AOR of 1.65 (95% CIs: 1.05–2.59, *P* = 0.031). After classifying SPB into SPL and PPROM, similar results were found in the first trimester; however, using multinomial logistic models, different results were found in the third trimester (Fig. 3 and Table S6). An increased risk of SPL was found for pregnant women with intermediate La exposure levels (AOR = 2.00, 95% CIs: 1.07–3.75, *P* = 0.030), high La exposure levels (AOR = 1.87, 95% CIs: 1.01–3.44, *P* = 0.045), and high Sm exposure levels (AOR = 1.82, 95% CIs: 1.00–3.30, *P* = 0.049) relative to the low exposure. We still found that La and Sm had a dose-response relationship with the risk of SPL (Table S6). Relative to the low exposure level, the AORs of the intermediate and high exposure levels were 2.16 and 2.00 for La, and 1.48 and 1.82 for Sm, respectively. For PPROM, an increased risk was found for pregnant women only with high Pr exposure levels (AOR = 1.69, 95% CIs: 1.00–2.85, P = 0.049). No dose-relationship of REE exposure levels with PPROM was found in adjusted multinomial models. After excluding pregnant women with a GBS infection, an increased risk of SPL was still found in high La exposure levels and high Sm exposure levels, and an increased risk of PPROM was still found in high Pr exposure levels (Table S7).

**Table 3:** Association between rare earth elements and SPB in the third trimester

Elements	Case	Control	OR	AOR
La				
low	74 (30.3)	89 (36.6)	1.00	1.00
intermediate	90 (36.9)	72 (29.6)	1.50 (0.97–2.33)	1.70 (1.07–2.68) *
high	80 (32.8)	82 (33.7)	1.17 (0.76–1.81)	1.28 (0.81–2.02)
*P*_trend			0.471	0.289
Ce				
low	79 (32.4)	84 (34.6)	1.00	1.00
intermediate	79 (32.4)	83 (34.2)	1.01 (0.66–1.56)	1.05 (0.67–1.64)
high	86 (35.2)	76 (31.3)	1.20 (0.78–1.86)	1.25 (0.80–1.96)
*P*_trend			0.405	0.327
Pr				
low	77 (31.6)	86 (35.4)	1.00	1.00
intermediate	70 (28.7)	92 (37.9)	0.85 (0.55–1.32)	0.88 (0.56–1.37)
high	97 (39.8)	65 (26.7)	1.67 (1.07–2.59) *	1.65 (1.05–2.59) *
*P*_trend			0.023*	0.033*
Nd				
low	81 (33.2)	82 (33.7)	1.00	1.00
intermediate	79 (32.4)	83 (34.2)	0.96 (0.62–1.49)	1.04 (0.66–1.62)
high	84 (34.4)	78 (32.1)	1.09 (0.71–1.68)	1.12 (0.71–1.75)
*P*_trend			0.698	0.626
Sm				
low	74 (30.3)	89 (36.6)	1.00	1.00
intermediate	89 (36.5)	73 (30.0)	1.47 (0.95–2.27)	1.43 (0.91–2.24)
high	81 (33.2)	81 (33.3)	1.20 (0.78–1.86)	1.23 (0.78–1.92)
P_trend			0.405	0.365
Eu				
low	147 (60.2)	151 (62.1)	1.00	1.00
high	97 (39.8)	92 (37.9)	1.08 (0.75–1.56)	1.11 (0.76–1.62)

^a^: Adjusted for ethnicity (Han & others), education level (Junior college and below, Undergraduate & Postgraduate and above), age (20–29, 30–34 & ≥35), BMI (slim, normal & overweight), parity (Primiparous & Multiparous), and family income (< 10,000, 10,000–19,999 & ≥20,000)

*: *P* < 0.05

**Fig. 4:**
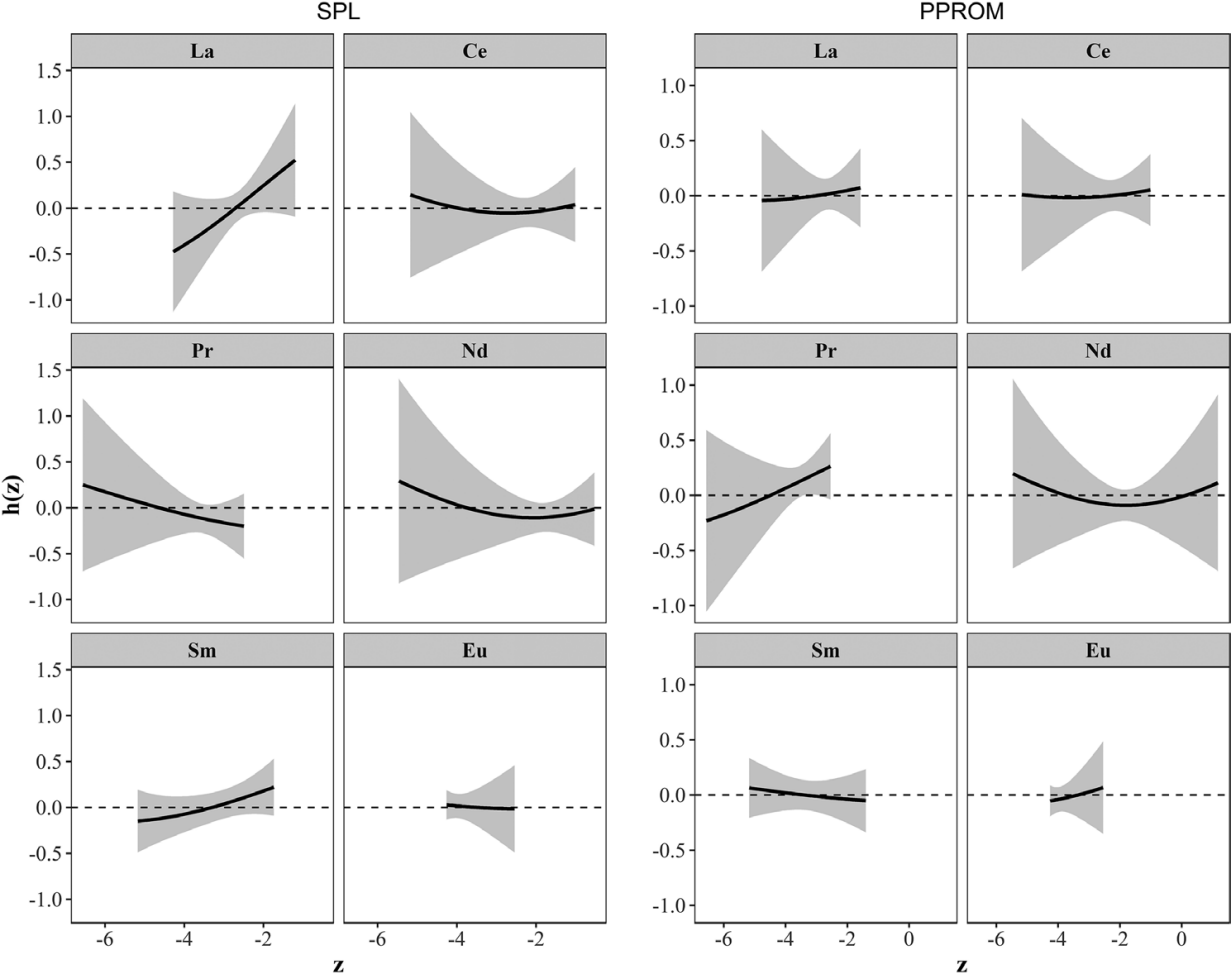
**Association between single rare earth element in the third trimester and SPL and PPROM.** Adjusted for ethnicity, education level, age, BMI, parity, and family income

**Fig. 5:**
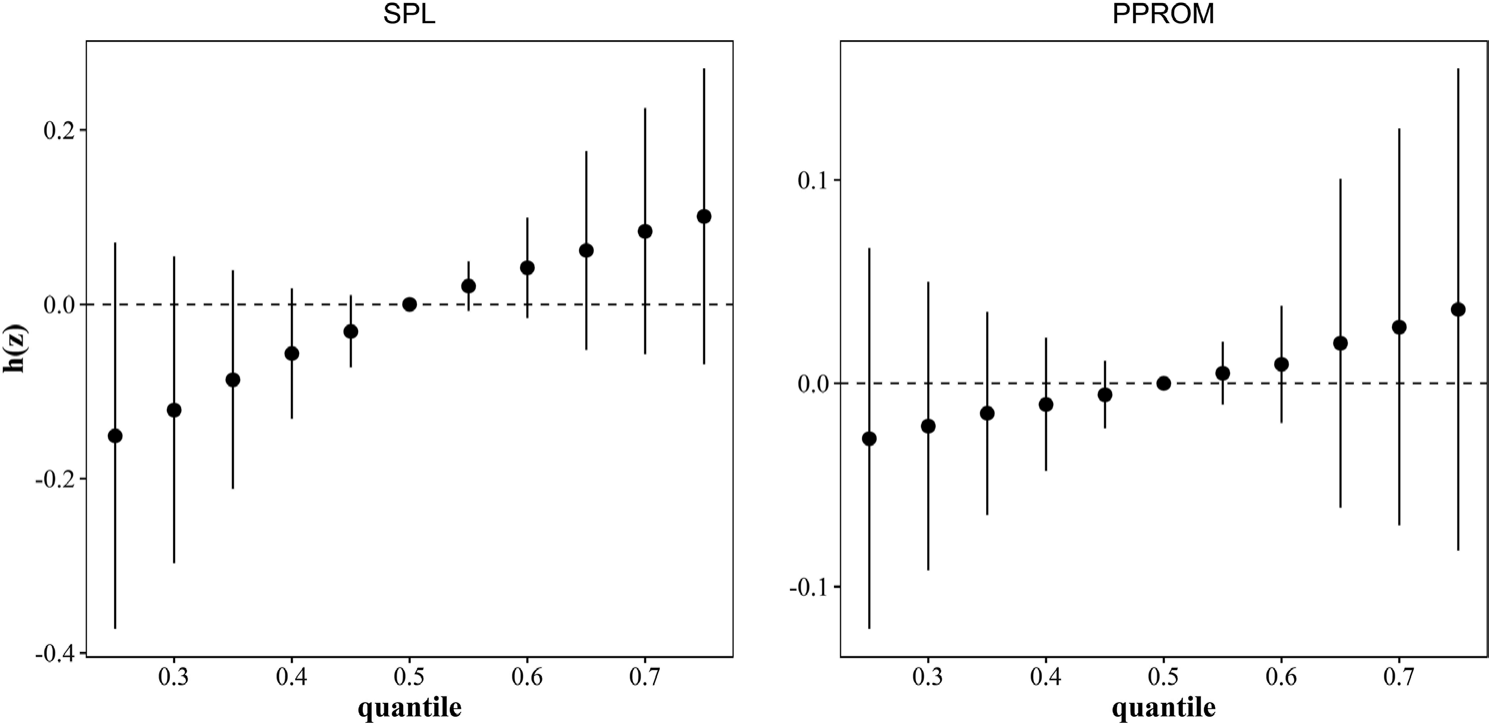
**Associations between mixed rare earth element exposures in the third trimester and SPL and PPROM.** Adjusted for ethnicity, education level, age, BMI, parity, and family income

Taking into account that the associations between REEs and SPL and PPROM were different and that no obvious association was found in the first trimester in former analyses, we used BKMR models only for SPL and PPROM in the third trimester. Figure 4 shows the exposure-response relationships between REEs and risks of SPL and PPROM in the third trimester. When other REEs were fixed at their median concentrations, and the models were adjusted for potential confounders, we found that La and Sm were positively associated with the risk of SPL, and Pr was positively associated with the risk of PPROM. Unlike the results in the multinomial models, Pr was negatively associated with the risk of SPL. Among all REEs, La (PIP = 0.72) and Pr (PIP = 0.34) contributed the most to SPL and PPROM, respectively (Table 4). When taking into account joint exposure effects, the increased risks of SPL and PPROM were associated with increased mixed REE exposure (Fig. 5). Considering the low detection rates of Eu, we excluded Eu in BKMR models to test the robustness of the results and the results did not change significantly (Figs. S1 and S2, Table S8). After excluding pregnant women with GBS infections, the results remained similar to the results obtained for all pregnant women (Figs. S3 and S4, Table S9).

**Table 4:** PIP of rare earth element exposure in the third trimester

REE	SPL	PPROM
La	0.72	0.29
Ce	0.45	0.29
Pr	0.39	0.34
Nd	0.47	0.26
Sm	0.48	0.17
Eu	0.39	0.31

## Discussion

Our study explored the associations between light REE exposure in different trimesters and SPB and its subtypes. We found that the associations between La, Pr, and Sm and SPB existed only in the third trimester. Different associations between light REE exposure and SPL and PPROM were found. In the third trimester, high La and Sm exposure levels were associated with elevated risk of SPL, and a high level of Pr exposure was associated with an elevated risk of PPROM, both including and excluding pregnant women with GBS infection.

REE exposure levels were highly correlated with local concentrations of REEs in environmental components [15]. However, the concentrations of REEs in our study were much lower than those in people who live around REE mines (Table S10) [38]. REE exposure levels vary among people living in non-mining areas and differ by region, age, and sex [19, 39]. A previous study found that all light REEs existed in 11 major food categories and the major REEs consumed from foods were La and Ce [40]. Another study suggested that traffic emissions could increase the concentrations of REEs in environmental components [16]. Different food habits and different family locations might lead to different REE exposure levels. A few previous studies have reported concentrations of REEs in pregnant women and newborns (Table S10). However, it is difficult to evaluate the relative exposure level between our study and other studies due to differences in sample types and populations. Only four studies have reported concentrations of REEs in serum or plasma samples of pregnant women. The concentrations of most REEs in our study were higher than those in pregnant women in Shanxi, China [21, 41]. With the exception of Sm and Eu, levels of REE exposure in our study are comparable to those in other pregnant women in Beijing, China [39]. However, our concentrations were lower than those in pregnant women in Serbia [42]. The types of REEs that are most abundant in any given area, and their translocation patterns, may differ by regions, which could explain these differences between studies. More studies are necessary to investigate REE exposure levels of pregnant women in China, considering that China has the greatest reserves of REEs and the wild range of REE applications in the world.

No previous studies have reported the potential associations between REE exposure and SPB. Liu et al. explored the potential associations between REEs in urine before delivery with premature rupture of membranes and PPROM [10]. Unlike other studies of preterm birth, they focused on the premature rupture of membranes, and the control group did not exclude women with preterm births. They found that high concentrations of REEs in urine (i.e., La, Ce, Pr, Nd, Eu, and some other heavy REEs) before delivery might be a risk factor for PPROM. Another study, which focused on preterm birth, found that Sm in cord blood was associated with the risk of preterm birth in the elastic net model but was not associated with gestational age or preterm birth in further analysis [43]. Taking into account the different causes of SPB and provider-initiated preterm birth, the results for preterm birth have limited interpretations for SPB. In addition, sample type, sampling trimester, and participants’ regions were also different between our study and the other two studies. These major differences could explain the different results among studies.

We found that La, Sm, and Pr exposure in the third trimester might increase the risk of SPL or PPROM. A previous study found that La exposure increased the risk of cessation of pregnancy and decreased litter sizes in mice [44]. REE exposure is usually associated with oxidative stress, inflammation, and cytogenetic effects [23, 45]. In previous animal experiments, exposure to La, Ce, and Nd increased the levels of malondialdehyde, reactive oxygen species, lipid peroxidation, and other biochemical indicators of oxidative stress [23, 46, 47]. This may have been, in part, a response to oxidative stress and inflammation, considering that these are detrimental factors in preterm birth pathology [26, 27]. Previous studies have also reported that SPL and PPROM were differ in terms of redox and other molecular processes [28, 48, 49]. This could potential explain the different effects of REE exposure on the different subtypes of SPB. However, in our study, relevant biomarkers were not measured, and further exploration could not be performed. Omics technology could be used to explore the differences in the effects of environmental exposure on SPL and PPROM in the future. Previous studies have also reported that REE exposure might increase the risk of neural tube defects, orofacial clefts, and failure of in vitro fertilization-embryo transfer [20–22]. More efforts are needed to monitor REE exposure levels and evaluate the health effects of REEs in pregnant women, particularly around REE mining areas in China.

To the best of our knowledge, this was the first study to explore the associations between REE exposure in the first and third trimesters and SPB and its subtypes. Previous studies have reported that any associations between elements and SPB might only exist in a specific trimester [6, 50]. Multiple measures of light REE exposure could be helpful to determine in which trimester light REE exposure might increase the risk of SPB. In addition, we explored associations between light REE exposure and subtypes of SPB, which may have different pathogeneses and susceptivity to environmental exposure [28, 29]. Further, 244 mothers who experienced SPB and who had plasma samples in the first and third trimesters were included in our study, a larger number than in previous studies on trace elements and SPB. Furthermore, excluding pregnant women with GBS infection could decrease the potential confounding effects of GBS infections on preterm birth. However, some limitations must be considered. First, although previous studies have shown that concentrations of REEs in plasma could reflect REE exposure levels, they generally reflect short-term rather than long-term exposure [51, 52]. Second, the gestational weeks of the control group were limited to between 39 and 41, which is not comparable to other studies of preterm birth. Births occurring between 37 and 0 days and 38 weeks 6 days were further subclassified as early term. Maternal and neonatal adverse outcome rates are higher for early-term births than for those occurring after 39 weeks [53, 54]. Thus, we only included births occurring between 39 and 41 weeks as our control groups. Third, it may not be possible to extrapolate our results to other populations because our study only included pregnant women living in Beijing, China. More studies in other places are needed to confirm our results. levels of REE exposure may be associated with different subtypes of SPB. Specifically, levels of La and Sm in plasma may be associated with SPL, and levels of Pr may be associated with PPROM. Multicenter and high-quality prospective studies are required to confirm our findings, particularly near areas of REE mining.

### Electronic supplementary material

Below is the link to the electronic supplementary material.

Supplementary Material 1

## Data Availability

The datasets used during the current study are available from the corresponding author on reasonable request.

## List of abbreviations

SPB: spontaneous preterm birth
SPL: spontaneous preterm labor
PPROM: preterm premature rupture of membranes
REEs: rare earth elements
La: lanthanum
Ce: cerium
Pr: praseodymium
Nd: neodymium
Pm: promethium
Sm: samarium
Eu: europium
ICP-MS: inductively coupled plasma-mass spectrometry
MDLs: method detection limits
OR: odds ratio
AOR: adjusted odds ratio
CIs: confidence intervals
BKMR: Bayesian kernel machine regression
PIP: Posterior inclusion probability
GBS: group B Streptococcus
