# Supplementary material for: Association between light rare earth elements in maternal plasma and the risk of spontaneous preterm birth: a nested case-control study from the Beijing birth cohort study
Source: Environ Health. 2023 Oct 23;22:73. doi: 10.1186/s12940-023-01027-1 (PMC10591387; doi:10.1186/s12940-023-01027-1)
Supplement: Supplementary file 1 — Supplementary Material 1 [file 12940_2023_1027_MOESM1_ESM.docx]

**Supplementary Materials:**

**Association between light rare earth elements in maternal plasma and the risk of spontaneous preterm birth: A nested case**-**control study from the Beijing Birth Cohort Study**

[**Text S1. Laboratory analysis.**](#_Toc147771077)

[**Table S1. The measured values and the reference concentrations of reference samples.**](#_Toc147771078)

[**Table S2. Method detection limits and the detection rates of rare earth elements.**](#_Toc147771079)

[**Table S3. Characteristics of pregnant women among different groups in Beijing, China, 2018-2020.**](#_Toc147771080)

[**Table S4. Comparison of median concentrations of rare earth elements among different groups.**](#_Toc147771081)

[**Table S5. Association between rare earth elements and SPB in the first trimester.**](#_Toc147771082)

[**Table S6. Association between levels of rare earth elements in the third trimester and SPL and PPROM.**](#_Toc147771083)

[**Table S7. Association between levels of rare earth elements in the third trimester and SPL and PPROM in pregnant women without GBS infection.**](#_Toc147771084)

[**Fig S1. Association between single rare earth element exposure in the third trimester and SPL/PPROM in pregnant women without Eu in models.**](#_Toc147771085)

[**Table S8. PIP of rare earth element exposure in the third trimester in pregnant women without Eu in models.**](#_Toc147771086)

[**Fig S2. Associations between mixed rare earth element exposure in the third trimester and SPL and PPROM in pregnant women Eu in models.**](#_Toc147771087)

[**Fig S3. Association between single rare earth element exposure in the third trimester and SPL/PPROM in pregnant women without GBS infection.**](#_Toc147771088)

[**Table S9. PIP of rare earth element exposure in the third trimester in pregnant women without GBS infection.**](#_Toc147771089)

[**Fig S4. Associations between mixed rare earth element exposure in the third trimester and SPL and PPROM in pregnant women without GBS infection.**](#_Toc147771090)

[**Table S10. Concentrations of rare earth elements in pregnant women or newborns in previous studies.**](#_Toc147771091)

## Text S1. Laboratory analysis.

The ICP-MS was operated at the following settings: gas flow, 0.96 L/min; auxiliary gas: 1.85 L/min; plasma gas: 17.5 L/min; radio frequency power: 1150 W; dwell time: 50-100 ms; resolution ratio: 0.7–0.9 amu; and single detected mode. The internal standard was ^187^Re and the isotope used were ^139^La, ^140^Ce, ^141^Pr, ^143^Nd, ^147^Sm, and ^153^Eu.

Eleven 1%(V:V) nitric acid were measured using 1%(V:V) nitric acid calibration based on within-run data to calculate the limits of detection. The instrument limits of detection were three times the standard deviation. The instrument limits of quantitation were three times the instrument limits of detection. The method limits of detection were the instrument limits of detection × dilution factor (i.e., 20), and the method limits of quantitation were the instrument limits of quantitation × dilution factor (i.e., 20).

## Table S1. The measured values and the reference concentrations of reference samples.

| Elements | Reference values(ng/g) | Measured value 1 | Measured value 2 | Measured value 3 | RSD (%) |
| --- | --- | --- | --- | --- | --- |
| ^139^La | 13.4±1.8 | 11.92 | 12.32 | 11.67 | 2.76 |
| ^140^Ce | 19.7±2.6 | 19.91 | 21.18 | 20.78 | 3.14 |
| ^141^Pr | (2.40) ^a^ | 2.52 | 2.61 | 2.39 | 4.49 |
| ^143^Nd | 8.4±1.5 | 8.71 | 8.47 | 8.82 | 2.05 |
| ^147^Sm | 1.4±0.4 | 1.52 | 1.56 | 1.67 | 4.76 |
| ^153^Eu | (0.60) ^a^ | 0.62 | 0.58 | 0.57 | 4.48 |

^a^: The reference values were provided by the National Standard Material Center in China with relatively low accuracy.

## Table S2. Method detection limits and the detection rates of rare earth elements.

| Elements ^a^ | Method detection  Limits ^b^(ng/mL) | Detection rates (n, %) | | Method quantitation  limits ^b^(ng/mL) | Rates of REEs≥ quantitation  limits (n, %) | |
| --- | --- | --- | --- | --- | --- | --- |
|  |  | Case | Control |  | Case | Control |
| La_1 | 0.012 | 244 (100.0) | 244 (100.0) | 0.008 | 234 (95.9) | 227 (93.0) |
| La_3 |  | 243 (99.6) | 243 (100.0) |  | 232 (95.1) | 221 (90.9) |
| Ce_1 | 0.008 | 244 (100.0) | 244 (100.0) | 0.006 | 244 (100.0) | 243 (99.6) |
| Ce_3 |  | 242 (99.2) | 243 (100.0) |  | 239 (98.0) | 239 (98.4) |
| Pr_1 | 0.002 | 244 (100.0) | 244 (100.0) | 0.001 | 240 (98.4) | 244 (100.0) |
| Pr_3 |  | 244 (100.0) | 242 (99.6) |  | 242 (99.2) | 240 (98.8) |
| Nd_1 | 0.006 | 243 (99.6) | 243 (99.6) | 0.004 | 239 (98.0) | 241 (98.8) |
| Nd_3 |  | 242 (99.2) | 243 (100.0) |  | 242 (99.2) | 241 (99.2) |
| Sm_1 | 0.008 | 215 (88.1) | 213 (87.3) | 0.006 | 175 (71.7) | 183 (75.0) |
| Sm_3 |  | 211 (86.5) | 209 (86.0) |  | 194 (79.5) | 179 (73.7) |
| Eu_1 | 0.02 | 61 (25.0) | 55 (22.5) | 0.014 | 0 (0.0) | 1 (0.4) |
| Eu_3 |  | 97 (39.8) | 92 (37.9) |  | 1 (0.4) | 1 (0.4) |

^a^: -1: first trimester; -3: third trimester.\

^b^: the calculation methods of the method detection limits and the method quantitation limits were shown in Text S1.

## Table S3. Characteristics of pregnant women among different groups in Beijing, China, 2018-2020.

| Characteristics ^a^ | Control (n=244) | SPL (n=99) | PPROM (n=145) | *P* ^b^ |
| --- | --- | --- | --- | --- |
| Age (year) |  |  |  |  |
| Mean±SD | 31.1±3.8 | 32.1±3.7 | 31.8±4.1 | 0.054 |
| 20–29 | 85 (34.8) | 26 (26.3) | 50 (34.5) | 0.145 |
| 30–34 | 111 (45.5) | 47 (47.5) | 54 (37.2) |  |
| ≥35 | 48 (19.7) | 26 (26.3) | 41 (28.3) |  |
| BMI (kg/m^2^) |  |  |  |  |
| Mean (SD) | 21.2±2.4 | 22.2±3.4 | 22.1±3.1 | 0.002 |
| Slim (BMI<18.5) | 29 (11.9) | 13 (13.1) | 16 (11.0) | 0.019 |
| Normal (18.5≤BMI<24) | 183 (75.0) | 61 (61.6) | 93 (64.1) |  |
| Overweight (BMI≥24) | 32 (13.1) | 25 (25.3) | 36 (24.8) |  |
| Ethnicity |  |  |  |  |
| Han | 226 (92.6) | 88 (88.9) | 135 (93.1) | 0.433 |
| Others | 18 (7.4) | 11 (11.1) | 10 (6.9) |  |
| Education level |  |  |  |  |
| Junior college and below | 49 (20.1) | 24 (24.2) | 42 (29.0) | 0.345 |
| Undergraduate | 140 (57.4) | 53 (53.5) | 70 (48.3) |  |
| Postgraduate and above | 55 (22.5) | 22 (22.2) | 33 (22.8) |  |
| Family income (yuan/month) |  |  |  |  |
| <10000 | 50 (20.6) | 23 (23.2) | 38 (26.2) | 0.305 |
| 10000–19999 | 92 (37.9) | 45 (45.5) | 51 (35.2) |  |
| ≥20000 | 101 (41.6) | 31 (31.3) | 56 (38.6) |  |
| Parity |  |  |  |  |
| Primiparous(= 1) | 186 (76.2) | 57 (57.6) | 100 (69.0) | 0.003 |
| Multiparous(≥2) | 58 (23.8) | 42 (42.4) | 45 (31.0) |  |
| GBS infection |  |  |  |  |
| Yes | 15 (6.1) | 3 (3.0) | 6 (4.1) | 0.470^c^ |
| No | 229 (93.9) | 96 (97.0) | 139 (95.9) |  |
| Infant sex |  |  |  |  |
| Male | 125 (51.2) | 60 (60.6) | 76 (52.4) | 0.275 |
| Female | 119 (48.8) | 39 (39.4) | 69 (47.6) |  |
| Sampling times (gestational weeks) |  |  |  |  |
| First trimester | 8.3±1.7 | 8.0±1.7 | 8.4±1.6 | 0.124 |
| Third trimester | 33.8±1.1 | 33.9±0.9 | 33.8±1.0 | 0.693 |

^a^: Data are presented as “mean±SD” or “number (percentage)”.

^b^: Pearson chi-square test or ANOVA test.

^c^: Fisher exact test.

## Table S4. Comparison of median concentrations of rare earth elements among different groups.

| Elements ^a^ | Control | SPL | PPROM | *P* ^b^ |
| --- | --- | --- | --- | --- |
| La-1 | 0.073 (0.058, 0.099) | 0.071 (0.058, 0.087) | 0.075 (0.060, 0.098) | 0.708 |
| La-3 | 0.070 (0.052, 0.088) | 0.075 (0.059, 0.094) | 0.069 (0.055, 0.086) | 0.217 |
| Ce-1 | 0.126 (0.091, 0.165) | 0.114 (0.081, 0.145) | 0.123 (0.088, 0.167) | 0.347 |
| Ce-3 | 0.102 (0.071, 0.134) | 0.108 (0.072, 0.147) | 0.102 (0.076, 0.139) | 0.234 |
| Pr-1 | 0.030 (0.024, 0.038) | 0.031 (0.023, 0.038) | 0.030 (0.022, 0.040) | 0.674 |
| Pr-3 | 0.030 (0.021, 0.037) | 0.031 (0.024, 0.041) | 0.032 (0.024, 0.039) | 0.111 |
| Nd-1 | 0.161 (0.115, 0.231) | 0.160 (0.111, 0.222) | 0.163 (0.116, 0.220) | 0.882 |
| Nd-3 | 0.173 (0.118, 0.234) | 0.188 (0.123, 0.232) | 0.170 (0.110, 0.249) | 0.453 |
| Sm-1 | 0.048 (0.024, 0.076) | 0.044 (0.018, 0.072) | 0.050 (0.024, 0.085) | 0.718 |
| Sm-3 | 0.049 (0.022, 0.091) | 0.063 (0.032, 0.099) | 0.050 (0.025, 0.088) | 0.355 |
| Eu-1 | 0.014 (0.014, 0.014) | 0.014 (0.014, 0.017) | 0.014 (0.014, 0.014) | 0.527 |
| Eu-3 | 0.014 (0.014, 0.023) | 0.014 (0.014, 0.025) | 0.014 (0.014, 0.027) | 0.485 |

^a^: Units: ng/mL; Data are presented as “median (quartile)”; -1: first trimester; -3: third trimester.

^b^: Statistical test by the Kruskal-Wallis test.

## Table S5. Association between rare earth elements and SPB in the first trimester.

| Elements ^a^ | Case | Control | OR | AOR^a^ |
| --- | --- | --- | --- | --- |
| La |  |  |  |  |
| low | 83 (34.0) | 80 (32.8) | 1.00 | 1.00 |
| intermediate | 82 (33.6) | 80 (32.8) | 0.99 (0.64–1.53) | 0.99 (0.63–1.55) |
| high | 79 (32.4) | 84 (34.4) | 0.91 (0.59–1.40) | 0.92 (0.59–1.45) |
| P_trend |  |  | 0.658 | 0.730 |
| Ce |  |  |  |  |
| low | 83 (34.0) | 80 (32.8) | 1.00 | 1.00 |
| intermediate | 83 (34.0) | 79 (32.4) | 1.01 (0.66–1.56) | 1.02 (0.65–1.60) |
| high | 78 (32.0) | 85 (34.8) | 0.88 (0.57–1.37) | 0.89 (0.57–1.39) |
| P_trend |  |  | 0.580 | 0.604 |
| Pr |  |  |  |  |
| low | 84 (34.4) | 79 (32.4) | 1.00 | 1.00 |
| intermediate | 78 (32.0) | 84 (34.4) | 0.87 (0.57–1.35) | 0.89 (0.57–1.40) |
| high | 82 (33.6) | 81 (33.2) | 0.95 (0.62–1.47) | 0.99 (0.63–1.55) |
| P_trend |  |  | 0.825 | 0.970 |
| Nd |  |  |  |  |
| low | 81 (33.2) | 82 (33.6) | 1.00 | 1.00 |
| intermediate | 79 (32.4) | 83 (34.0) | 0.96 (0.62–1.49) | 1.03 (0.66–1.61) |
| high | 84 (34.4) | 79 (32.4) | 1.08 (0.70–1.66) | 1.09 (0.70–1.71) |
| P_trend |  |  | 0.740 | 0.696 |
| Sm |  |  |  |  |
| low | 80 (32.8) | 83 (34.0) | 1.00 | 1.00 |
| intermediate | 78 (32.0) | 84 (34.4) | 0.96 (0.62–1.49) | 1.00 (0.64–1.57) |
| high | 86 (35.2) | 77 (31.6) | 1.16 (0.75–1.79) | 1.14 (0.73–1.80) |
| P_trend |  |  | 0.506 | 0.557 |
| Eu |  |  |  |  |
| low | 183 (75.0) | 189 (77.5) | 1.00 | 1.00 |
| high | 61 (25.0) | 55 (22.5) | 1.15 (0.75–1.74) | 1.06 (0.69–1.63) |

^a^: Adjusted for ethnicity (Han & others), education level (Junior college and below, Undergraduate & Postgraduate and above), age (20–29, 30–34 & ≥35), BMI (slim, normal & overweight), parity (Primiparous & Multiparous), and family income (<10000, 10000–19999 & ≥20000).

*: *P*<0.05.

##

## Table S6. Association between levels of rare earth elements in the third trimester and SPL and PPROM.

|  | SPL | | PPROM | |
| --- | --- | --- | --- | --- |
| Elements ^a^ | OR | AOR | OR | AOR |
| La |  |  |  |  |
| low | 1.00 | 1.00 | 1.00 | 1.00 |
| intermediate | 1.73 (0.95–3.15) | **2.00 (1.07–3.75)*** | 1.39 (0.85–2.28) | 1.54 (0.92–2.57) |
| high | 1.69 (0.94–3.04) | **1.87 (1.01–3.44)*** | 0.91 (0.54–1.52) | 0.98 (0.58–1.67) |
| P_trend | 0.081 | **0.049*** | 0.758 | 0.983 |
| Ce |  |  |  |  |
| low | 1.00 | 1.00 | 1.00 | 1.00 |
| intermediate | 0.89 (0.50–1.59) | 0.88 (0.48–1.59) | 1.10 (0.66–1.83) | 1.17 (0.70–1.98) |
| high | 1.14 (0.65–2.00) | 1.20 (0.67–2.15) | 1.25 (0.75–2.08) | 1.28 (0.76–2.16) |
| P_trend | 0.659 | 0.553 | 0.386 | 0.353 |
| Pr |  |  |  |  |
| low | 1.00 | 1.00 | 1.00 | 1.00 |
| intermediate | 0.66 (0.36–1.20) | 0.66 (0.36–1.23) | 1.00 (0.60–1.66) | 1.03 (0.61–1.74) |
| high | 1.60 (0.91–2.79) | 1.58 (0.88–2.81) | **1.72 (1.03–2.87)*** | **1.69 (1.00–2.85)*** |
| P_trend | 0.107 | 0.130 | **0.040*** | 0.054 |
| Nd |  |  |  |  |
| low | 1.00 | 1.00 | 1.00 | 1.00 |
| intermediate | 1.23 (0.69–2.18) | 1.37 (0.75–2.48) | 0.82 (0.49–1.36) | 0.86 (0.51–1.44) |
| high | 1.23 (0.69–2.21) | 1.29 (0.70–2.36) | 1.01 (0.62–1.66) | 1.02 (0.61–1.70) |
| P_trend | 0.491 | 0.420 | 0.975 | 0.942 |
| Sm |  |  |  |  |
| low | 1.00 | 1.00 | 1.00 | 1.00 |
| intermediate | 1.50 (0.82–2.74) | 1.48 (0.80–2.76) | 1.45 (0.88–2.37) | 1.39 (0.84–2.31) |
| high | 1.73 (0.97–3.08) | **1.82 (1.00–3.30)*** | 0.92 (0.55–1.53) | 0.92 (0.54–1.57) |
| P_trend | 0.059 | **0.046*** | 0.795 | 0.827 |
| Eu |  |  |  |  |
| low | 1.00 | 1.00 | 1.00 | 1.00 |
| high | 1.26 (0.78–2.03) | 1.32 (0.80–2.15) | 0.97 (0.64–1.49) | 0.99 (0.64–1.54) |

^a^: Adjusted for ethnicity, education level, age, BMI, parity, and family income.

*: *P*<0.05.

## Table S7. Association between levels of rare earth elements in the third trimester and SPL and PPROM in pregnant women without GBS infection.

|  | SPL | | PPROM | |
| --- | --- | --- | --- | --- |
| Elements ^a^ | OR | AOR | OR | AOR |
| La |  |  |  |  |
| low | 1.00 | 1.00 | 1.00 | 1.00 |
| intermediate | 1.69 (0.91–3.14) | 1.88 (0.99–3.60) | 1.26 (0.76–2.10) | 1.35 (0.80–2.29) |
| high | 1.74 (0.95–3.18) | **1.88 (1.00–3.54)*** | 0.87 (0.52–1.47) | 0.92 (0.53–1.57) |
| P_trend | 0.077 | 0.055 | 0.635 | 0.764 |
| Ce |  |  |  |  |
| low | 1.00 | 1.00 | 1.00 | 1.00 |
| intermediate | 0.75 (0.42–1.37) | 0.74 (0.40–1.36) | 0.97 (0.58–1.64) | 1.02 (0.60–1.74) |
| high | 1.09 (0.61–1.92) | 1.11 (0.62–2.01) | 1.20 (0.71–2.01) | 1.21 (0.71–2.05) |
| P_trend | 0.779 | 0.726 | 0.491 | 0.489 |
| Pr |  |  |  |  |
| low | 1.00 | 1.00 | 1.00 | 1.00 |
| intermediate | 0.71 (0.39–1.31) | 0.70 (0.37–1.32) | 1.03 (0.61–1.75) | 1.05 (0.62–1.79) |
| high | 1.73 (0.98–3.08) | 1.69 (0.94–3.07) | **1.77 (1.04–3.00) *** | **1.72 (1.00–2.94) *** |
| P_trend | 0.059 | 0.081 | **0.036*** | 0.054 |
| Nd |  |  |  |  |
| low | 1.00 | 1.00 | 1.00 | 1.00 |
| intermediate | 1.33 (0.73–2.42) | 1.45 (0.78–2.68) | 0.83 (0.49–1.39) | 0.85 (0.50–1.44) |
| high | 1.26 (0.69–2.29) | 1.31 (0.70–2.45) | 0.88 (0.53–1.48) | 0.90 (0.53–1.51) |
| P_trend | 0.479 | 0.416 | 0.632 | 0.677 |
| Sm |  |  |  |  |
| low | 1.00 | 1.00 | 1.00 | 1.00 |
| intermediate | 1.49 (0.80–2.77) | 1.49 (0.79–2.83) | 1.30 (0.79–2.16) | 1.26 (0.75–2.12) |
| high | **1.87 (1.03–3.38) *** | **1.96 (1.06–3.63) *** | 0.91 (0.54–1.54) | 0.91 (0.53–1.57) |
| P_trend | **0.037*** | **0.030*** | 0.759 | 0.784 |
| Eu |  |  |  |  |
| low | 1.00 | 1.00 | 1.00 | 1.00 |
| high | 1.29 (0.80–2.09) | 1.38 (0.83–2.28) | 0.89 (0.58–1.38) | 0.92 (0.59–1.45) |

^a^: Adjusted for ethnicity, education level, age, BMI, parity, and family income.

*: *P*<0.05.


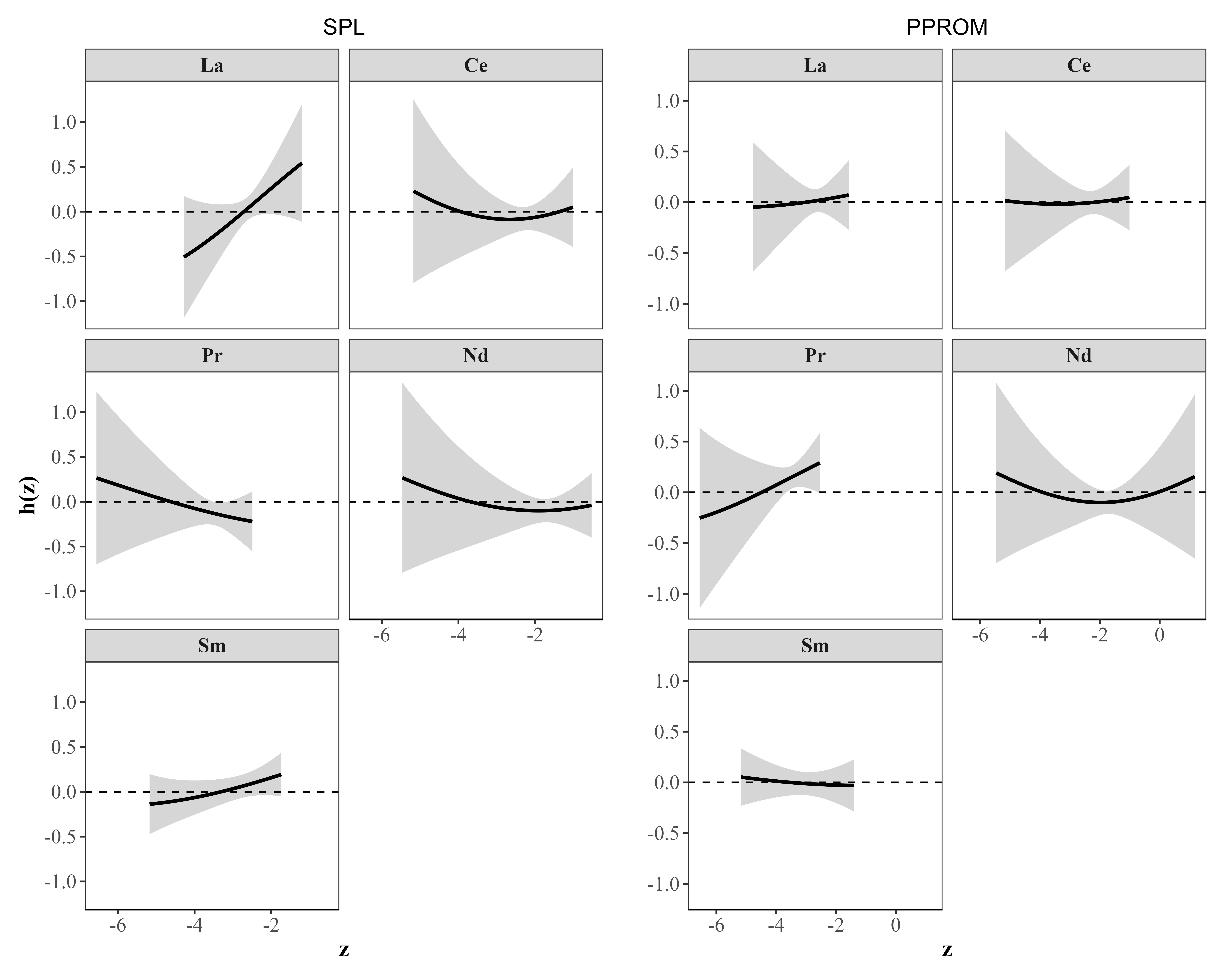


## Fig S1. Association between single rare earth element exposure in the third trimester and SPL/PPROM in pregnant women without Eu in models. Adjusted for ethnicity, education level, age, BMI, parity, and family income.

## Table S8. PIP of rare earth element exposure in the third trimester in pregnant women without Eu in models.

| REEs | SPL | PPROM |
| --- | --- | --- |
| La | 0.76 | 0.34 |
| Ce | 0.45 | 0.33 |
| Pr | 0.40 | 0.38 |
| Nd | 0.43 | 0.32 |
| Sm | 0.44 | 0.21 |


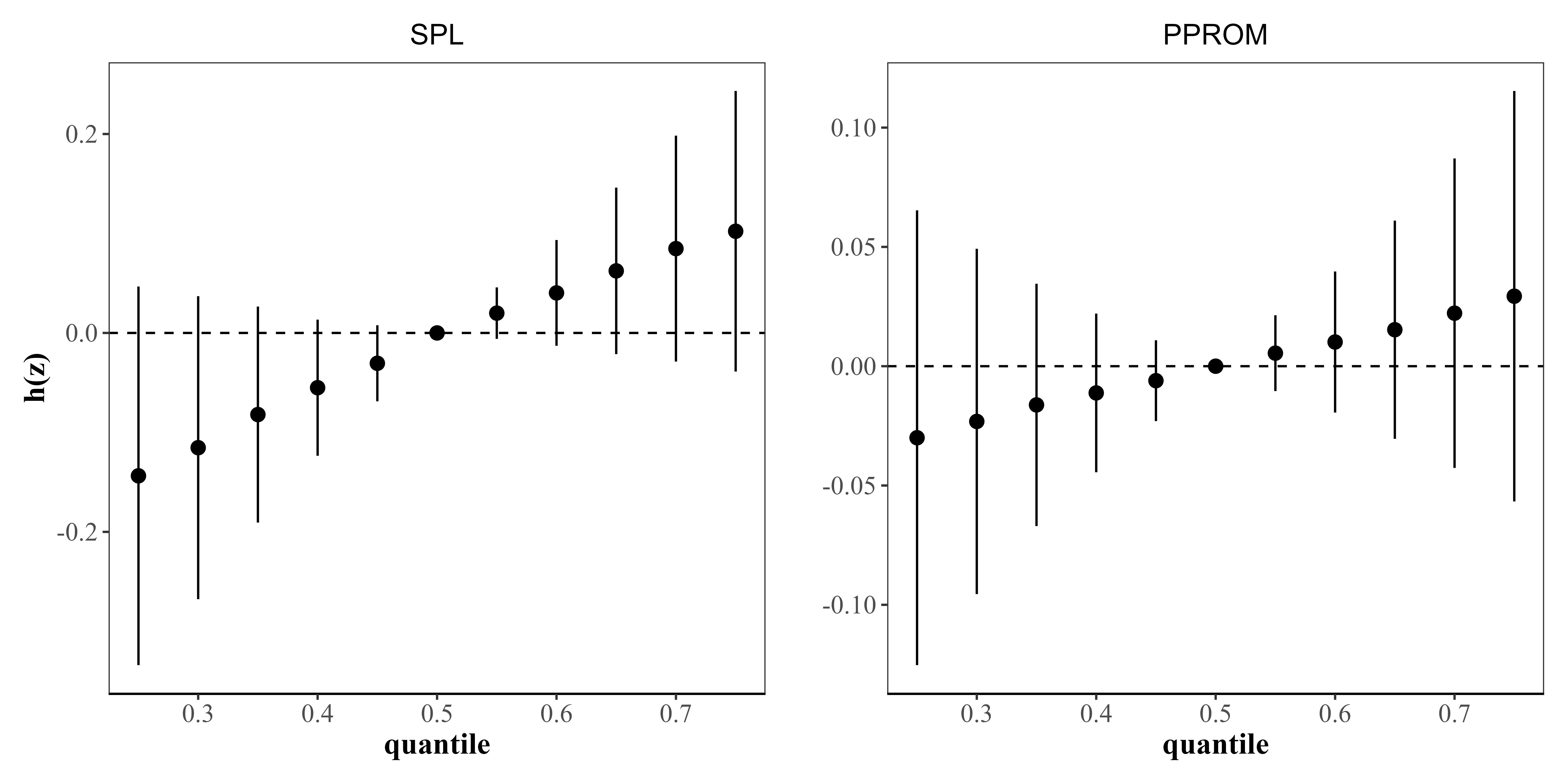


## Fig S2. Associations between mixed rare earth element exposure in the third trimester and SPL and PPROM in pregnant women Eu in models. Adjusted for ethnicity, education level, age, BMI, parity, and family income.


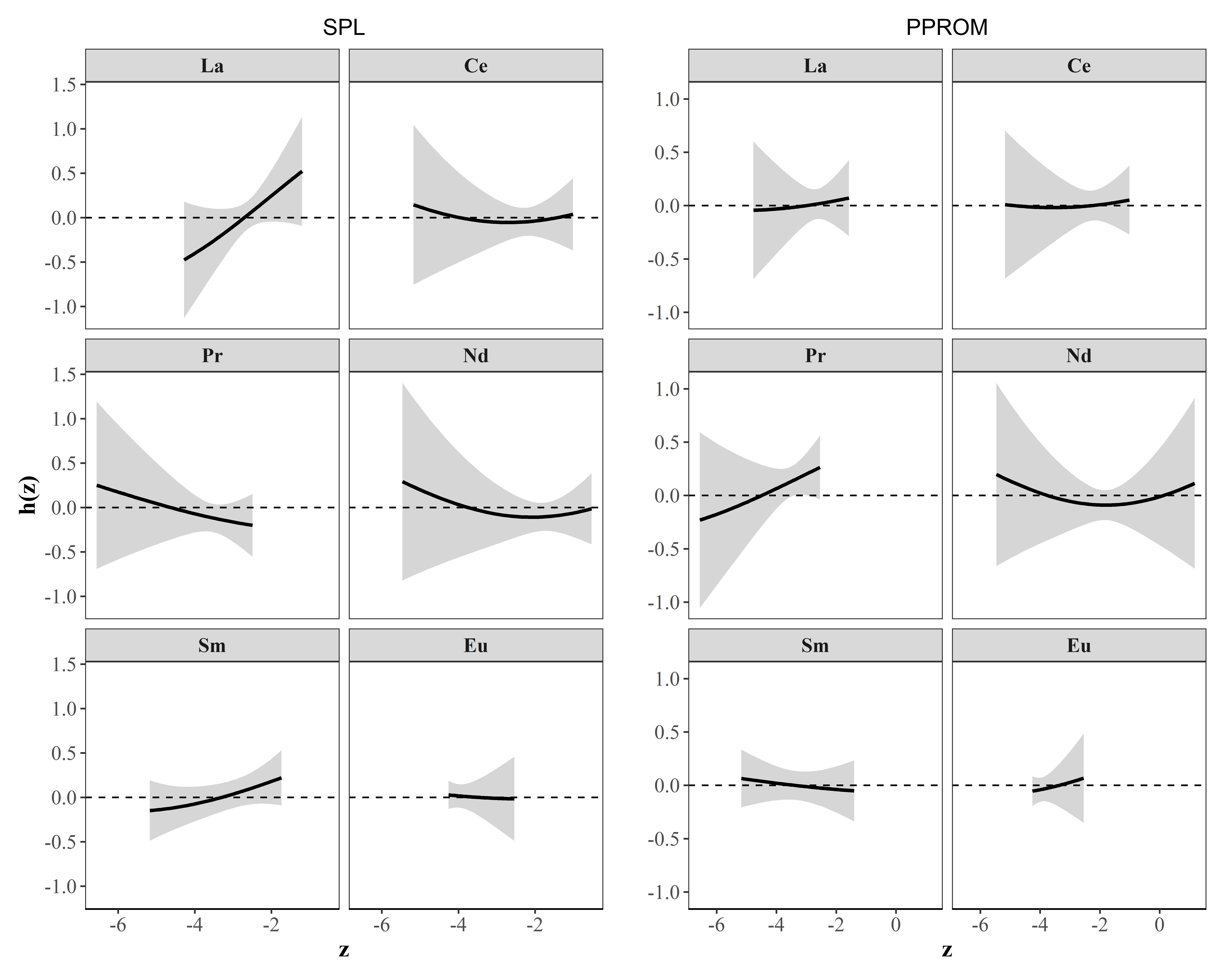


## Fig S3. Association between single rare earth element exposure in the third trimester and SPL/PPROM in pregnant women without GBS infection. Adjusted for ethnicity, education level, age, BMI, parity, and family income.

## Table S9. PIP of rare earth element exposure in the third trimester in pregnant women without GBS infection.

| REEs | SPL | PPROM |
| --- | --- | --- |
| La | 0.73 | 0.35 |
| Ce | 0.45 | 0.37 |
| Pr | 0.37 | 0.45 |
| Nd | 0.46 | 0.36 |
| Sm | 0.47 | 0.26 |
| Eu | 0.37 | 0.36 |


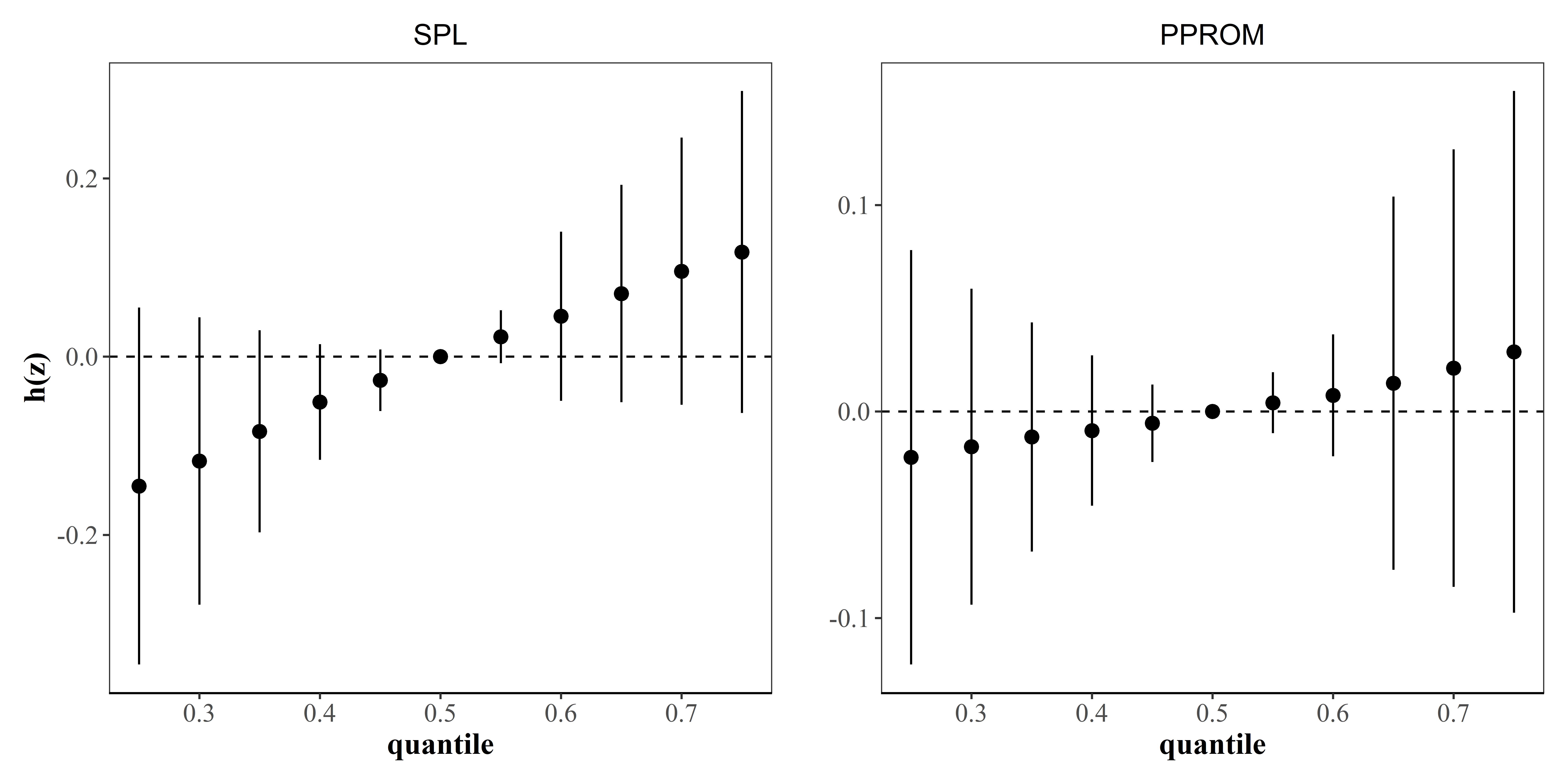


## Fig S4. Associations between mixed rare earth element exposure in the third trimester and SPL and PPROM in pregnant women without GBS infection. Adjusted for ethnicity, education level, age, BMI, parity, and family income.

## Table S10. Concentrations of rare earth elements in pregnant women or newborns in previous studies.

| References | Region | Sample type (ng/mL) | Sampling time | Subgroup | La | Ce | Pr | Nd | Sm | Eu |
| --- | --- | --- | --- | --- | --- | --- | --- | --- | --- | --- |
| This study | Beijing, China | Plasma | First trimester | / | 0.073 ^a^  (0.059, 0.096) | 0.121  (0.088, 0.162) | 0.031  (0.023, 0.039) | 0.162  (0.114, 0.225) | 0.047  (0.022, 0.077) | <MDL  (<MDL, <MDL) |
|  |  |  | Third trimester | / | 0.071  (0.054, 0.088) | 0.103  (0.072, 0.138) | 0.030  (0.023, 0.039) | 0.176  (0.116, 0.238) | 0.053  (0.025, 0.093) | <MDL  (<MDL, 0.025) |
| (Xu et al., 2021) | Beijing, China | Serum | First trimester | / | 0.076 (0.054, 0.099) | 0.137 (0.091, 0.179) | 0.029 (0.023, 0.035) | 0.178 (0.134, 0.226) | 0.123^c^ (0.092, 0.164) | 0.027  (<MDL, 0.036) |
| (Wei et al., 2020) | Shanxi, China | Serum | / | Cases | 0.072  (0.052, 0.107) | 0.116  (0.080, 0.233) | 0.030  (0.023, 0.043) | 0.212  (0.156, 0.265) | 0.132  (0.098, 0.173) | 0.034  (0.023, 0.048) |
|  |  |  |  | Controls | 0.059  (0.045, 0.083) | 0.090  (0.063, 0.130) | 0.028  (0.022, 0.037) | 0.195  (0.150, 0.252) | 0.127  (0.096, 0.168) | 0.030  (0.022, 0.042) |
| (Yan. et al., 2020) | Shanxi, China | Serum | First trimester | / | 0.047  (0.036, 0.061) | 0.066  (0.049, 0.090) | 0.020  (0.016, 0.024) | 0.125  (0.103, 0.155) | 0.094  (0.076, 0.116) | 0.019  (0.015, 0.024) |
|  |  |  | Second trimester | / | 0.049  (0.038, 0.066) | 0.069  (0.056, 0.093) | 0.021  (0.017, 0.025) | 0.140  (0.109, 0.173) | 0.094  (0.078, 0.115) | 0.020  (0.015, 0.024) |
| (Stojsavljević et al., 2022) | Serbia | Plasma | Beginning of the delivery | / | 0.67±0.24 ^b^ | 0.17±0.12 | 0.25±0.09 | 0.78±0.32 | 0.33±0.16 | 0.14±0.08 |
|  |  | Umbilical cord plasm |  | / | 0.091±0.077 | 0.16±0.08 | 0.059±0.028 | 0.16±0.09 | 0.12±0.06 | 0.040±0.027 |
| (Liu et al., 2021b) | Hubei, China | Urine (ug/g creatinine) | Before delivery |  | 0.066  (0.027, 0.149) | 0.065  (0.027, 0.149) | 0.015  (0.006, 0.036) | 0.043  (0.021, 0.094) | / | 0.015  (0.006, 0.042) |
| (Liu et al., 2019) | Hubei, China | Urine (ug/g creatinine) | Before delivery |  | / | 0.065  (0.026, 0.151) | / | / | / | / |
| (Liu et al., 2021a) | Shanxi, China | Umbilical cord tissue (ng/g) | / | Cases | 0.70  (0.46, 1.11) | 6.17  (2.56, 18.71) | 0.18  (0.10, 0.34) | 0.56  (0.38, 0.94) | 0.21  (0.11, 0.70) | 0.01  (<MDL, 2.60) |
|  |  |  |  | Controls | 0.49  (0.23, 0.93) | 6.64  (2.28, 17.5) | 0.14  (0.08, 0.24) | 0.45  (0.20, 0.82) | 0.17  (0.04, 0.81) | 0.01  (<MDL, 2.60) |
| (Cabrera-Rodriguez et al., 2018) | Canary Islands, Spain | umbilical cord blood | / | / | 0.01  (0.01, 0.03) | 0.03  (0.02, 0.06) | <MDL  (<MDL, 0.01) | 0.01  (<MDL, 0.01) | <MDL  (<MDL, 0.01) | 0.01  (<MDL, 0.02) |
| (Huang et al., 2021) ^a^ | Bangladesh | umbilical cord blood | / | / | 0.056 | 5.536 | 0.013 | 0.039 | 0.015 | 0.010 |
| (Stojsavljević et al., 2021) | Caucasus | placental tissues (ng/g) | / | / | 0.41  (0.25, 0.61) | 0.42  (0.27, 0.80) | 0.043  (0.029, 0.061) | 0.135  (0.087, 0.196) | 0.010  (0.006, 0.020) | 0.021  (0.014, 0.029) |
| Reference 38 in the manuscript ^d^ | Bao tou, China | Whole blood | Non-pregnant women | / | 0.854  (0.702, 1.061) | 1.724  (1.446, 2.498) | 0.132  (0.110, 0.165) | 0.839  (0.587, 0.815) | / | / |

^a^: Data are presented as “median (quartile)”.

^b^: Data are presented as “mean ± SD” or “mean”.

^c^: Data in red means concentration was higher than this study and in blue means lower than this study.

^d^: Data was shown for readability because reference 38 in the manuscript were in Chinese.

**References**

Cabrera-Rodriguez, R., Luzardo, O.P., Gonzalez-Antuna, A., Boada, L.D., Almeida-Gonzalez, M., Camacho, M., Zumbado, M., Acosta-Dacal, A.C., Rial-Berriel, C., Henriquez-Hernandez, L.A., 2018. Occurrence of 44 elements in human cord blood and their association with growth indicators in newborns. Environ Int 116, 43-51. doi:10.1016/j.envint.2018.03.048

Huang, H., Wei, L., Chen, X., Zhang, R., Su, L., Rahman, M., Golam Mostofa, M., Qamruzzaman, Q., Zhao, Y., Yu, H., Wei, Y., Christiani, D.C., Chen, F., 2021. Cord serum elementomics profiling of 56 elements depicts risk of preterm birth: Evidence from a prospective birth cohort in rural Bangladesh. Environ Int 156, 106731. doi:10.1016/j.envint.2021.106731

Liu, L., Wang, L., Ni, W., Pan, Y., Chen, Y., Xie, Q., Liu, Y., Ren, A., 2021a. Rare earth elements in umbilical cord and risk for orofacial clefts. Ecotoxicol Environ Saf 207, 111284. doi:10.1016/j.ecoenv.2020.111284

Liu, Y., Wu, M., Song, L., Bi, J., Wang, L., Chen, K., Liu, Q., Xiong, C., Cao, Z., Li, Y., Xia, W., Xu, S., Wang, Y., 2021b. Association between prenatal rare earth elements exposure and premature rupture of membranes: Results from a birth cohort study. Environ Res 193, 110534. doi:10.1016/j.envres.2020.110534

Liu, Y., Wu, M., Zhang, L., Bi, J., Song, L., Wang, L., Liu, B., Zhou, A., Cao, Z., Xiong, C., Yang, S., Xu, S., Xia, W., Li, Y., Wang, Y., 2019. Prenatal exposure of rare earth elements cerium and ytterbium and neonatal thyroid stimulating hormone levels: Findings from a birth cohort study. Environ Int 133, 105222. doi:10.1016/j.envint.2019.105222

Stojsavljević, A., Rovčanin, M., Miković, Ž., Perović, M., Jeremić, A., Zečević, N., Manojlović, D., 2022. Analysis of essential, toxic, rare earth, and noble elements in maternal and umbilical cord blood. Environ Sci Pollut Res Int 29, 37375-37383. doi:10.1007/s11356-021-18190-y

Stojsavljević, A., Rovčanin, M., Rovčanin, B., Miković, Ž., Jeremić, A., Perović, M., Manojlović, D., 2021. Human biomonitoring of essential, nonessential, rare earth, and noble elements in placental tissues. Chemosphere 285, 131518. doi:10.1016/j.chemosphere.2021.131518

Wei, J., Wang, C., Yin, S., Pi, X., Jin, L., Li, Z., Liu, J., Wang, L., Yin, C., Ren, A., 2020. Concentrations of rare earth elements in maternal serum during pregnancy and risk for fetal neural tube defects. Environ Int 137, 105542. doi:10.1016/j.envint.2020.105542

Xu, X., Wang, Y., Han, N., Yang, X., Ji, Y., Liu, J., Jin, C., Lin, L., Zhou, S., Luo, S., Bao, H., Liu, Z., Wang, B., Yan, L., Wang, H.J., Ma, X., 2021. Early Pregnancy Exposure to Rare Earth Elements and Risk of Gestational Diabetes Mellitus: A Nested Case-Control Study. Front Endocrinol (Lausanne) 12, 774142. doi:10.3389/fendo.2021.774142

Yan., H., Yan., L., Zhang., L., Zhang., Y., Pang., Y., Hao., Y., Wang., B., Li., Z., Rongwei Ye, 2020. The association between serum light rare earth elements in pregnant women and small for gestational age birth [in Chinese]. Chinese Journal of Reproductive Health 31, 501-505+511.
